# Factors associated with onset‐age in major affective disorders

**DOI:** 10.1111/acps.13497

**Published:** 2022-09-15

**Authors:** Alessandro Miola, Leonardo Tondo, Paola Salvatore, Ross J. Baldessarini

**Affiliations:** ^1^ International Consortium for Mood & Psychotic Disorders Research, Mailman Research Center McLean Hospital Belmont Massachusetts USA; ^2^ Department of Psychiatry University of Padova Padua Italy; ^3^ Department of Psychiatry Harvard Medical School Boston Massachusetts USA; ^4^ Lucio Bini Mood Disorder Centers Cagliari Rome Italy; ^5^ Center for Healthcare Organization & Implementation Research US Veterans Administration Medical Center Bedford Massachusetts USA

**Keywords:** age at onset, bipolar disorder, major depression, morbidity

## Abstract

**Background:**

Research findings on factors associated with onset‐age (OA) with bipolar (BD) and major depressive disorders (MDD) have been inconsistent, but often indicate greater morbidity following early OA.

**Methods:**

We considered factors associated with OA in 1033 carefully evaluated, systematically followed mood disorder subjects with DSM‐5 BD (*n* = 505) or MDD (*n* = 528), comparing rates of descriptive and clinical characteristics following early (age <18), intermediate (18–40), or later onset (≥40 years), as well as regressing selected measures versus OA. Exposure time (years ill) was matched among these subgroups.

**Results:**

As hypothesized, many features were associated with early OA: familial psychiatric illness, including BD, greater maternal age, early sexual abuse, nondepressive first episodes, co‐occurring ADHD, suicide attempts and violent suicidal behavior, abuse of alcohol or drugs, smoking, and unemployment. Other features increased consistently with later OA: %‐time‐depressed (in BD and MDD, women and men), as well as depressions/year and intake ratings of depression, educational levels, co‐occurring medical disorders, rates of marriage and number of children.

**Conclusions:**

OA averaged 7.5 years earlier in BD versus MDD (30.7 vs. 38.2). Some OA‐associated measures may reflect maturation. Associations with family history and suicidal risk with earlier OA were expected; increases of time‐depressed in both BD and MDD with later OA were not. We conclude that associations of OA with later morbidity are complex and not unidirectional but may be clinically useful.

**Significant outcomes**

- OA averaged 7.5 years earlier among patients diagnosed with bipolar disorder (BD) than with major depressive disorder (MDD).
- Associated with *earlier* OA were familial psychiatric illness, early sexual abuse; nondepressive first episodes, attention disorder, substance abuse, and suicidal acts.
- Depressive first episodes, %‐time‐depressed and depressions/year, as well as siblings, education, marriage, children, and general medical comorbidity all increased with *later* onset.

**Limitations**

- Recall bias may affect ascertainment of OA and details of early illness.
- Some effects may merely reflect maturation and the passage of time.

## 1 INTRODUCTION

Age‐at‐onset (OA) of illness has been proposed as a useful indicator of relatively homogeneous subgroups of patients with mood and other psychiatric disorders. Its potential value includes support of early differentiation of bipolar (BD) from major depressive disorders (MDD), prediction of the nature and severity of long‐term morbidity, and improved separation of familial or potentially genetically based, from sporadic or more environmentally influenced mood disorders, as well as supporting timely interventions with specifically targeted treatments.^1^, ^2^, ^3^, ^4^, ^5^

A comprehensive review of research studies found a pooled value for OA in BD of 25.3 years (95% confidence interval [CI]: 24.4–26.2),^6^ whereas MDD had a broad range of OA with a somewhat later average of 30 years in some studies,^7^, ^8^ but as low as 24.8 [CI: 17.2–36.0] years,^9^ and with an even younger average OA for MDD than for BD (30 vs. 33 years) in one worldwide study.^8^ In BD, in particular, OA may not follow a simple unimodal distribution, but instead can yield bimodal^10^, ^11^, ^12^ or trimodal distributions.^13^, ^14^, ^15^, ^16^, ^17^

Although subjects initially presenting with a hypomanic or a manic episode are easily diagnosed as having BD, an unresolved clinical challenge has been to differentiate subjects presenting with a depressive episode who eventually may meet criteria for BD. Although early identification of these cases has been difficult, in some cases it is possible to predict development of a BD if there is a family history for BD, early OA, and a high number of depressive episodes, as well as early evidence of impulsivity or of mixed manic‐depressive features.^18^ In such cases, it is prudent to consider treatment appropriate for BD and to use antidepressants cautiously. Lack of recognition of previous episodes of mania and especially of hypomania can contribute to frequent delay by several years to establish a BD diagnosis and initiate appropriate treatment, and such delay may affect outcome adversely.^19^, ^20^, ^21^

Early‐onset BD specifically has been associated with greater illness severity, including psychotic features,^15^, ^22^, ^23^, ^24^, ^25^, ^26^, ^27^ more mixed episodes,^14^, ^15^, ^16^, ^23^, ^25^, ^26^, ^28^ and greater risk of substance abuse^15^, ^27^, ^28^, ^29^, ^30^, ^31^ as well as co‐occurring panic and obsessive–compulsive disorders.^15^, ^16^, ^23^, ^31^ Early‐onset MDD also has been linked to unfavorable long‐term outcomes, including greater risk of recurrences,^32^, ^33^, ^34^, ^35^ impaired social function,^36^ and higher prevalence of psychotic^37^ as well as atypical features.^37^, ^38^, ^39^ Early‐onset MDD is also reported to carry increased risk of co‐occurring anxiety and substance abuse disorders,^35^, ^38^, ^39^, ^40^, ^41^, ^42^ more co‐occurring general medical disorders,^35^ higher suicidal risks,^35^, ^39^, ^41^, ^42^, ^43^ and greater familial loading for depression.^41^, ^43^, ^44^, ^45^

Nevertheless, such associations have been inconsistent, including of earlier‐onset mood disorders with family history and with particular forms or severity of later morbidity.^6^, ^14^, ^30^, ^37^, ^38^, ^46^, ^47^ In addition, distinguishing older adults with BD versus MDD by early OA may have limited clinical usefulness.^48^, ^49^, ^50^

To address unresolved questions about relationships between OA in both BD and MDD, we carried out extensive analyses of relationships of descriptive and clinical characteristics including morbidity with OA, both in pre‐defined early (<18), intermediate (18–40), and later (>40 years) OA subgroups as well as with OA as a continuous measure. We also considered possible differences with DSM‐5 diagnoses of BD versus MDD and among women versus men. Based on the research just reviewed, we hypothesized that earlier OA would be associated with family history of mood disorders and with more severe morbidity in later years in both BD and MDD as well as in both women and men.

## 2 METHODS

### 2.1 Subjects

The study sample was a large cohort of consecutive adult participants evaluated and followed at the Lucio Bini Mood Disorders Centers in Cagliari, Sardinia and Rome, Italy, which are specialized outpatient clinics for affective disorder patients. Subjects were clinically diagnosed with a major affective disorder (BD‐1 or BD‐2, MDD), updated to DSM‐5 criteria, and underwent systematic initial and repeated diagnostic evaluations during follow‐up, using semi‐structured interviews by the same mood disorder expert (LT), leading to construction of individual life‐charts. Onset age (OA) was defined as a first syndromal psychiatric illness followed by sufficient evidence to support a DSM‐5 diagnosis of BDD or MDD. OA was determined retrospectively from repeated interviews of patients, verified by family members, based on age at any first lifetime apparently syndromal psychiatric illness followed by diagnosis of BD or MDD during long‐term, systematic follow‐up. A few subjects (8.2%) were evaluated at the study sites at first episodes. First episodes also were identified from medical records of mental health professionals or primary‐care clinicians or at psychiatric hospitalization prior to referral to the study sites. Participants provided written informed consent at clinical entry for potential anonymous reporting of clinical data in aggregate analyses, in accordance with requirements of Italian law.

### 2.2 Clinical measures

We assessed information about current and lifetime psychiatric diagnoses, family history in first‐degree relatives, course of illness after onset, rates of suicidal ideation or acts, substance abuse, illness episodes/year, and best estimates of %‐time ill overall or in depression or [hypo]mania based on life‐charts, as well as associated medical and psychiatric conditions. Severity of depressive symptoms was assessed at clinic intake using the 21‐item Hamilton Depression Rating Scale (HDRS_21_),^51^ and [hypo]manic symptoms with the Young Mania rating Scale (YMRS)^52^ and the Mood Disorder Questionnaire (MDQ).^53^ Affective temperaments were rated with the 39‐item version of the self‐assessment TEMPS‐A scale.^54^

### 2.3 Data analysis

Data are presented as means with 95% confidence intervals (CI). Sociodemographic and selected clinical data were analyzed with OA as a continuous measure as well as in pre‐defined *early* (<18), *intermediate* (18–40), and *later* (>40 years) OA subgroups, using contingency tables (*χ* ^2^ or Fisher's exact test) for categorical measures, analysis of variance (*t*‐test) for continuous measures. To avoid potential artifacts of different exposure times in OA subgroups (increased rates with shorter times or later OA, more chance to observe categorical outcomes with longer time‐at‐risk or earlier OA), we matched years of illness to the shortest exposure time (latest onset). These subgroup comparisons were tabulated as ratios (and their CIs) of magnitudes of differences at the extreme OAs (<18 vs. >40). We also used multivariable linear regression modeling to identify selected factors that were significantly and independently associated with OA as a continuous measure. Statistical significance was limited to guiding selection of initial measures for further analyses, generally considering two‐tailed *p* < 0.005 to indicate particularly interesting findings and compensate for multiple comparisons. Analyses employed commercial software: *Statview.5* (SAS Institute, Cary, NC) for spreadsheets, and *Stata.1*7 (StataCorp, College Station, TX) for analyses.

## 3 RESULTS

### 3.1 Characteristics associated with onset‐age

The total of 1033 consecutive, consenting adult subjects diagnosed with a DSM‐5 major affective disorder included 505 with BD (258 BD‐I; 247 BD‐II) and 528 with MDD; 632 (61.2%) were women and 401 (38.8%) men, and current age averaged 47.3 years [46.2–48.3]. Subjects were ill a total of 12.7 years [CI: 12.2–13.2] (matched across OA groups to the shortest exposure with oldest onset) and were followed systematically at the study sites for an average of 4.43 years [4.16–4.70]. Onset‐age (OA) averaged 34.5 years [CI: 33.5–35.5] overall and was 7.50 years younger in BD (30.7 [29.5–31.9]; BD‐I: 27.6 [26.2–29.0]; BD‐II: 33.9 [32.1–35.7]) than MDD (38.2 [36.8–39.6]).

Consistent with the initial hypothesis that many characteristics of subjects with mood disorders would differ by OA, we found consistently *decreasing* values of several factors among subjects with older OA (greater with earlier OA). These included: young (<18) > intermediate (18–40) > older (>40 years) OA for the following *descriptive* outcome measures (Table 1): [a] mean onset‐age (by definition); [b] older age of mother at subject's birth (with a similar but weaker trend for father's age); [c] first‐lifetime illness episodes characterized by mania or hypomania (“[hypo]mania”), mixed manic‐depressive episodes, psychosis, or anxiety disorders rather than depression; [d] measures of familial risk of psychiatric illnesses among first‐degree relatives, including the rate (%) of affected persons among relatives, overall and with both BD and MDD subjects, as well as risk of having relatives diagnosed with BD (but not MDD) specifically; [e] early sexual (but not physical) abuse; [f] risk of being unemployed; [g] higher ratings with the Mood Disorder Questionnaire (MDQ) with its emphasis on lifetime [hypo]manic features, but not YMRS mania scores at intake.

**TABLE 1:** Characteristics of mood‐disorder subjects associated with early, intermediate, and late onset ages

Measure^a^	Onset‐age groups (years) [means with 95% CI]			*χ* ^2^ or *t*‐score	*p*‐Value
	<18	18–40	>40		
*Subjects* (*n* [%])				—	—
BD	74 [63.8%]	303 [54.0%]	128 [36.0%]		
MDD	42 [36.2%]	258 [46.0%]	228 [64.0%]		
Total	116 [100%]	561 [100%]	356 [100%]		
*Mean onset‐age* (yrs)					
BD	15.2 [14.7–15.9]	26.3 [25.7–27.0]	50.1 [48.7–51.5]	31.0	<0.0001
MDD	15.1 [13.4–16.9]	27.6 [26.9–28.3]	54.5 [53.1–55.9]	29.2	<0.0001
Total	15.2 [14.5–15.5]	26.9 [26.4–27.4]	52.9 [51.9–53.9]	42.9	<0.0001
Current age	28.0 [25.9–30.0]	39.6 [38.8–40.4]	65.7 [64.5–66.8]	31.8	<0.0001
Exposure (years of illness)	12.7 [11.5–13.9]	12.7 [12.1–13.3]	12.7 [11.7–13.7]	0.02	0.99
Women (%)	43.1 [33.9–52.6]	60.4 [56.2–64.5]	68.3 [63.1–73.1]	23.6	<0.0001
Mother's age at subject's birth	32.9 [31.1–34.7]	30.6 [29.6–31.6]	29.1 [28.0–30.2]	2.38	0.004
*First episode type* (%)				105	<0.0001
Depressed	61.2 [51.7–70.1]	64.7 [60.6–68.7]	87.6 [83.7–90.8]		
[Hypo]manic, mixed, psychotic	13.8 [8.09–21.4]	20.6 [17.3–24.2]	8.17 [5.54–11.5]		
Anxious	15.5 [9.46–23.4]	13.6 [10.9–16.7]	3.66 [1.96–6.18]		
Other	9.48 [4.83–16.3]	1.08 [0.40–2.33]	0.56 [0.07–2.02]		
*Family history* (%)					
Overall	34.1 [31.4–36.9]	25.9 [24.4–27.4]	20.8 [12.8–22.7]	5.46	<0.0001
in BD	33.8 [30.4–37.1]	25.4 [23.6–27.2]	20.0 [16.9–23.1]	4.22	<0.0001
in MDD	35.0 [30.0–40.0]	26.8 [24.2–29.3]	21.4 [18.8–23.9]	3.55	<0.0001
% of Relatives (rate)	32.0 [26.0–38.0]	28.5 [25.9–31.2]	21.9 [19–24.6.2]	2.66	0.0009
BD in family	27.6 [23.5–32.0]	21.9 [19.8–24.1]	19.4 [16.3–22.8]	10.2	0.006
Siblings (*n*/subject)	1.68 [1.29–2.06]	2.34 [2.13–2.55]	3.52 [3.19–3.86]	5.47	<0.0001
Early sexual abuse (%)	32.4 [18.0–49.8]	11.3 [6.74–17.5]	9.88 [4.36–18.5]	12.6	0.002
Educated >high school	6.54 [2.67–13.0]	22.9 [19.1–27.0]	25.7 [20.7–31.3]	17.5	0.0002
*Intake ratings*					
HDRS: overall	14.8 [13.2–16.4]	16.8 [16.2–17.5]	18.6 [17.9–19.2]	3.66	<0.0001
BD	14.4 [12.2–16.4]	15.5 [14.6–16.4]	18.3 [17.1–19.5]	2.72	0.0007
MDD	15.4 [13.1–17.8]	18.3 [17.5–19.0]	18.7 [17.9–19.5]	2.18	0.009
MDQ	9.70 [8.04–11.3]	9.18 [8.30–10.1]	6.41 [5.57–7.64]	3.11	0.0001
*Marital history*					
Ever married (%)	8.70 [4.25–15.4]	43.9 [39.7–48.1]	72.8 [67.8–77.4]	160	<0.0001
Children/subject	0.09 [0.42–0.56]	0.86 [0.92–1.14]	1.85 [1.83–2.07]	9.29	<0.0001
Unemployed (%)	16.5 [10.1–24.8]	9.95 [7.58–12.7]	1.78 [0.65–3.82]	32.2	<0.0001

*Note*: Other first episodes included attention, substance abuse and eating disorders; family history is the % of subjects with any psychiatric illness in one or more first‐degree relatives, or rate (% of relatives). Measures not significantly different or consistently increasing or decreasing among onset age groups include: population density where raised, family history of suicide, age of father at birth of patient, early physical abuse, socioeconomic status (SES), intake ratings of [hypo]mania (YMRS), functioning (GAF), affective temperament types (TEMPS‐A), marital separations, and body‐mass index (BMI, kg/m^2^).

Abbreviations: BD, bipolar disorder; HDRS, 21‐item Hamilton depression rating scale; MDD, major depressive disorder; MDQ, mood disorder questionnaire.

In contrast, other factors became *more* likely or prominent with older OA (Table 1): [a] greater number of siblings (which may reflect the passage of time); [b] major depressive initial illness episode; [c] higher depression score (HDRS) at intake with both BD and MDD; [d] greater likelihood of marriage; and [e] more children per subject.

### 3.2 Psychiatric morbidity and onset‐age

Risk or rates of some morbidity factors *declined* with older OA: [a] % of time in [hypo]mania; [b] rates of suicide attempts and of violent suicidal acts; [c] substance abuse (overall and for alcohol, cannabis and tobacco smoking, as well as poly‐substance abuse); [d] co‐occurring attention disorder (ADHD). Factors that *increased* with later OA included: [a] co‐occurring general medical disorders; [b] as well as a striking and unexpected finding that the proportion of time at risk involving depression rose consistently with older OA overall, as well as with both BD and MDD, and among women and men; [c] there was a similar but weaker increase in the rate of depressions/year (Table 2).

**TABLE 2:** Morbidity following early, intermediate, and late onset‐ages in 1033 major affective disorder subjects

Measure	Onset‐age groups (years) [means with 95% CI]			*χ* ^2^ or *t‐*score	*p*‐Value
	<18	18–40	>40		
*Depression*					
% Time depressed					
Overall	20.3 [17.0–23.6]	22.0 [20.3–23.8]	29.0 [26.4–31.6]	3.55	<0.0001
With MDD	22.1 [14.4–29.8]	20.9 [16.9–24.8]	33.5 [28.9–38.2]	3.07	0.0001
With BD	19.8 [16.1–23.5]	22.4 [20.8–24.4]	25.6 [22.7–28.5]	1.72	0.05
In women	21.6 [16.6–26.6]	21.3 [19.1–23.4]	28.3 [25.2–35.0]	2.74	0.0006
In men	19.5 [15.1–23.9]	23.2 [20.2–26.2]	30.2 [25.4–31.4]	2.41	0.003
Depressions/year	0.49 [0.42–0.57]	0.52 [0.47–0.57]	0.65 [0.53–0.77]	1.79	0.04
% Time [Hypo]manic	7.74 [5.54–9.94]	7.47 [6.43–8.51]	4.55 [3.70–5.40]	2.72	0.0007
*Suicidal status* (%)					
Attempts	27.0 [19.1–36.0]	15.4 [12.5–18.7]	10.8 [7.75–14.5]	17.1	<0.0001
Violent acts	13.0 [5.37–24.9]	5.16 [2.51–8.73]	1.88 [0.45–6.27]	11.4	0.003
*Substance abuse* (%)					
Any	31.9 [21.1–28.4]	16.5 [10.8–13.6]	2.91 [2.64–5.10]	70.3	<0.0001
Tobacco smoking	62.3 [49.8–73.7]	57.2 [51.1–63.2]	37.8 [30.8–45.2]	38.9	<0.0001
Ethanol	28.0 [16.8–27.3]	9.09 [7.41–12.3]	4.00 [1.46–6.52]	12.2	0.0004
Polyabuse	45.6 [32.4–59.3]	26.3 [20.5–32.9]	5.10 [1.67–11.5]	11.1	0.0005
Cannabis	17.5 [17.3–24.2]	9.76 [8.92–11.6]	2.04 [1.23–3.09]	6.92	0.01
*Co‐occurring* (%)					
Medical disorders	23.4 [13.8–35.7]	51.2 [44.8–57.7]	85.5 [79.4–90.4]	90.4	<0.0001
ADHD	33.8 [22.8–46.3]	23.2 [18.3–28.6]	15.6 [10.2–22.3]	9.35	0.009

*Note*: Measures that did not differ significantly or did not rise or fall consistently across onset‐age groups included: episodes/year, total %‐time ill, [hypo]mania/year, rapid or continuous cycling course, hospitalizations/year, cigarettes/day, and cups of coffee/day.

### 3.3 Summary of factors associated with onset‐age

Highlights of descriptive, clinical or morbidity measures that were particularly strongly associated with earlier or later OA is provided with ranking by magnitude of the contrast in risk in the youngest versus oldest OA groups and its variance

### 3.4 Risk of depression versus [hypo]mania with onset age

**FIGURE 1:**
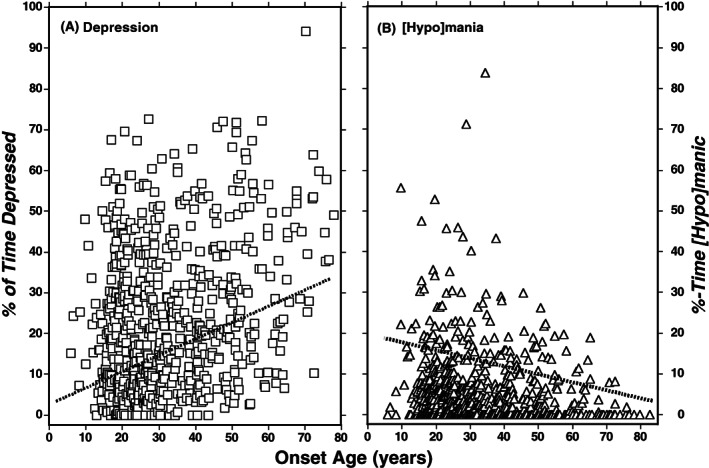
(A) Percentage of time at risk in depression versus age at onset of major affective disorders in 1033 subjects with matched exposure times (years at risk). Slope = 0.302 [95% CI: 0.215–0.390], *t*‐score = 6.77, *p* < 0.0001. (B) Percentage of time at risk in mania or hypomania versus onset age. Slope [95% CI] = −0.0009 [−0.0014 to −0.0005], *t* = 3.91, *p* = 0.0001. Note that there was more depression and less [hypo]mania with older illness onset.

We carried out linear regression modeling of associations of affective morbidity versus OA as a continuous measure (Figure 1). Consistent with other measures (Table 2), the findings indicated a highly significant rise in the proportion of time at risk in depression (A) and more moderate but significant decline of the proportion of time at risk in [hypo]mania (B).

### 3.5 Multivariate linear regression modeling

Several factors remained significantly and independently associated with OA as a continuous measure (Table 4). These ranked: more children, more co‐occurring general medical disorders, greater %‐time in depression, more MDD versus BD, greater likelihood of marriage, less substance abuse, and more women than men. Notably, however, exposure time was not associated with OA.

## 4 DISCUSSION

This study involved data collected longitudinally at the study‐sites over an average of 4.43 years regarding 1033 consecutive adult subjects diagnosed with a DSM‐5 major affective disorder (505 with BD and 528 with MDD) who were evaluated and systematically followed by a single mood disorder expert applying consistent assessment procedures. We examined the relationship of OA to descriptive and clinical characteristics including types and amounts of morbidity. OA was significantly younger in BD versus MDD patients and also significantly younger in BD‐1 than BD‐2. We considered OA in pre‐defined early (<18), intermediate (18–40), and later (>40 years) subgroups, as well as evaluating effects of OA as a continuous measure. The present analyses yielded several noteworthy results. First, OA averaged 7.5 years earlier among subjects diagnosed with DSM‐5 BD versus MDD, ranking: BD‐1 < BD‐2 < MDD, as we found previously,^4^ and in accord with a large international epidemiological survey, in which the OA for BD was 6 years younger than with MDD.^55^ Several other factors also were more likely with *earlier* OA (Tables 1, 2, 3).

**TABLE 3:** Differences between early and late onset mood disorders

Factor	Onset age (years)		Ratio [95% CI]
	<18	>40	
*Greater with earlier onset (<18/>40]*			
Substance abuse (%)	31.9	2.91	11.0 [9.83–12.3]
Unemployed (%)	16.5	1.78	9.27 [8.07–10.7]
Poly‐substance abuse (%)	45.6	5.10	8.94 [8.24–9.72]
Violent suicide attempt (%)	13.0	1.88	6.91 [6.06–7.94]
Early sexual abuse (%)	32.4	9.88	3.28 [3.11–346]
Suicide attempt (%)	27.0	10.8	2.50 [2.17–2.93]
Attention disorder (ADHD, %)	33.8	15.6	2.17 [1.94–2.45]
%‐Time [hypo]manic	7.74	4.55	1.70 [1.61–1.81]
First episode [hypo]manic, mixed, psychotic (%)	13.8	8.17	1.69 [1.62–1.78]
Any psychiatric family history (%)	34.1	20.8	1.64 [1.51–1.80]
Mother's age at patient birth	32.9	29.1	1.13 [1.09–1.18]
Exposure (years)	12.7	12.7	1.00 [0.97–1.03]
*Greater with later onset [>40/<18]*			
Children (*n*)	0.09	1.85	20.6 [10.7–44.5
Ever married (%)	8.70	72.8	8.37 [6.88–10.3]
Educated >high school (%)	6.54	25.7	3.98 [2.50–3.56]
Co‐occurring medical disorders (%)	23.4	85.5	3.65 [3.28–4.10]
Siblings (*n*)	1.63	2.34	2.10 [1.32–15.8]
Rapid or continuous cycling (%)	5.81	15.1	1.60 [2.44–2.78]
%‐Women	43.1	68.3	1.58 [1.56–1.76]
% Time depressed	20.3	29.0	1.43 [1.33–1.55]
First episode depression (%)	61.2	87.6	1.43 [1.37–1.50]
Depressions/year	0.49	0.65	1.33 [1.17–1.58]
Intake depression (HDRS)	14.8	18.6	1.26 [1.17–1.37]
% of Affected relatives	21.0	21.9	1.04 [1.02–1.08]

*Note*: Rates by onset age‐groups are from Tables 1 and 2 (all at *p* < 0.005), except for exposure.

Some of these associations may merely reflect the passage of more time after early OA (such as number of siblings) or contributions of maturation and aging (including marriage, more children, and co‐occurring general medical disorders). Others, however may be characteristic of an early‐onset type of mood disorders, notably with higher rates of affected first‐degree relatives and a specific family history of BD, early sexual abuse, first‐lifetime episodes other than depression, co‐occurring attention disorder (ADHD), higher intake ratings of lifetime bipolar characteristics (MDQ), less education, more unemployment, various types of substance abuse and smoking.

Initial depression was associated with older OA (Table 1), and there was a higher proportion of time in depression as well as somewhat greater rate of depressive recurrences and less time in [hypo]manic episodes with increasing OA (Tables 2, 3, 4 and Figure 1). The tendency toward more depression with older OA was also associated with greater risk of depressive first‐episodes, and higher ratings of depressive symptoms (HDRS scores) at intake to the study centers. These associations may reflect the tendency for BD to present earlier than MDD, as found in the present analyses. This interpretation also appears to accord with previous research on clinical factors that tended to validate the diagnosis of BD and its distinction from MDD, including younger onset.^56^, ^57^, ^58^, ^59^, ^60^ There also is growing evidence that the clinical characteristics of major depression can change with older current age, including a generally more severe course, longer episodes with slower recovery, and more co‐occurring general medical disorders, but less substance abuse or suicide attempts, following a possibly older OA and consequent shorter time ill.^61^, ^62^, ^63^

**TABLE 4:** Multivariable linear regression model of factors associated with later onset age in major affective disorder patients

Factor	Slope (*β*) [95% CI]	*t*‐Score	*p*‐Value
More children	3.91 [2.94–4.89]	7.88	<0.0001
More general medical disorders	7.44 [5.10–9.78]	6.26	<0.0001
Greater %‐time depressed	5.24 [3.94–7.75]	6.02	<0.0001
More MDD, less BD	5.99 [3.60–8.38]	4.93	<0.0001
Married	5.96 [3.26–8.67]	4.33	<0.0001
Less substance abuse	4.60 [1.76–7.45]	3.18	0.002
More women	2.70 [1.00–5.01]	2.30	0.02

*Note*: Factors tested including those with ratio of onset <18 versus >40 years ≥2.0 in Table 3, plus sex, diagnosis, and years at risk. Years at risk was unrelated to onset age.

The association of earlier OA with co‐occurring ADHD agrees with growing evidence of such an association and that ADHD can be an antecedent of mood disorders.^64^, ^65^ Previous investigations found that BD subjects with co‐occurring ADHD had a 5–6‐year earlier average OA than those without ADHD^64^, ^66^ and that among MDD subjects with ADHD, OA was approximately 10 years earlier than without ADHD.^66^

Also in accord with the present findings, childhood maltreatment has been associated with increased vulnerability to psychiatric illnesses generally and specifically with earlier OA of major mood disorders.^67^, ^68^, ^69^ Interestingly, the present findings provide evidence supporting the hypothesis that childhood trauma may mediate the association between family history of mood disorder and mood disorder in adulthood.^70^ The present findings also are consistent with previous reports of higher rates of substance abuse^15^, ^27^, ^28^, ^29^, ^30^, ^31^, ^35^, ^38^, ^40^, ^41^ as well as suicidal behavior^14^, ^15^, ^16^, ^25^, ^26^, ^28^, ^29^, ^35^, ^39^, ^41^, ^42^, ^43^ in early‐onset BD and MDD. An uncommon finding is the association of earlier OA with older maternal age at the time of the subject's birth and a weaker tendency toward older fathers, as has been noted previously.^71^

Other factors were associated with *later* OA (Tables 1 and 2). The higher proportion of women may represent a trait, whereas greater likelihood of marriage and of more children, higher education and more employment may reflect the availability of more time to reach important personal accomplishments before the onset of illness as well as adverse effects of early illness. The increase of time in depression with later OA follows depressive first‐lifetime episodes and higher intake ratings of depressive symptoms (HDRS) in both BD and MDD, as well as somewhat more depressions/year and fewer [hypo]manias/year. The rising %‐of‐time depressed with later OA was found in both BD and MDD, and in women and men, with matching of exposure time to avoid artifacts that may be associated with shorter time‐at‐risk after later OA.

Increased time to recovery from late‐life depression has been associated with greater severity of depressive symptoms, as might be expected, and late OA was a particularly strong predictor of slow recovery.^72^ A Netherlands study of the two‐year course of late‐life depression identified more severe depression and more somatic comorbidity as independent determinants of inferior clinical outcome of depression, and that those with younger OA were more likely to be depressed at follow‐up.^73^

The effect of greater density of depressive morbidity with *later* onset in both BD and MDD is unexpected, based on literature reviewed above. Also noteworthy is that the proportion of time spent in depression was nearly six‐times greater than in [hypo]mania (averaging 23.8%/3.98% across OA subgroups; Table 2). This outcome may well reflect responses to clinically determined treatments, which are much more effective against [hypo]mania in BD than against depression in MDD or BD.^74^

Also of note, the increased intensity of depression after older onset appears to accord with an excess of depressive first‐episodes with older OA as well as relatively high ratings for depressive symptoms (but not for mania) at intake, with a corresponding decrease in the proportion of time in [hypo]mania with older OA (Figure 1). Also possible is an effect of the presence of BD2 subjects with their great excess of depressive morbidity,^74^ whose OA (33.9 years) was intermediate between BD1 (27.6) and MDD cases (38.2). This possibility is supported by multivariable regression modeling of %‐time depressed against diagnosis [BD2 vs BD1] and OA, both of which were strongly and independently related to depression (not shown; slope = 5.86 [CI: 3.95–11.3] for OA, 5.11 [3.01–7.21] for diagnosis [both *t* ≥ 4.07, both *p* < 0.0001]). It may also be that the start of major mood disorders following more maturation, accomplishments, and expectations can contribute to greater risk of depression thereafter as a manifestation of the impact of loss following previous personal gains. In addition, some neurodegenerative disorders are preceded by more than a decade by depression.^75^, ^76^ However, the greater risk of depressive first episodes with more depression later suggests that less reactive causal factors, or vulnerabilities also may be at work.^77^

Potential clinical value of the present findings includes the possibility of predicting later types and amounts of morbidity, most notably, greater risk of depression and of diagnosis of MDD versus BD, but less risk of substance abuse and suicidal behavior, as well as greater academic and personal accomplishment with increasing onset‐age (Table 3).

## 5 LIMITATIONS

Limitations of this study include risk of recall bias pertaining to OA and to details of early illness by subjects or their relatives. However, such information often was supported by clinical records, and 8.2% of study participants were evaluated prospectively at the study sites from their first episode of illness. Nevertheless, any ascertainment errors were likely to be distributed randomly across OA and diagnosis. Moreover, post‐onset morbidity was verified in most cases by prolonged follow‐up at the study sites.

## 6 CONCLUSIONS

OA averaged 7.5 years earlier in BD than MDD (30.7 vs. 38.2). Potential effects of exposure‐time (longer with earlier OA) were limited by matching for years of illness. Some OA‐associated measures may reflect maturation and the passage of time. Overall, time in [hypo]mania was six‐times less than in depression, possibly as a manifestation of the relative efficacy of clinically employed treatments. Early OA was associated with greater suicidal risk and more substance abuse. Greater family history associated with early OA may reflect a higher genetic load, whereas substance abuse and other co‐occurring psychiatric syndromes may contribute to risk of later mood disorders. The proportion of time spent in [hypo]mania declined somewhat with older OA, but time in depression increased greatly overall, and in both BD and MDD and among both women and men. Some of the present findings, including OA itself, may help to guide timely differentiation of BD from MDD and to predict later morbidity. In general, the present findings indicate that associations of OA with later morbidity are strong, complex, and not unidirectional.

## CONFLICT OF INTEREST

The authors declare no conflict of interest.

### PEER REVIEW

The peer review history for this article is available at https://publons.com/publon/10.1111/acps.13497.

## Data Availability

The data that support the findings of this study are available on request from the corresponding author. The data are not publicly available due to privacy or ethical restrictions.
